# Comparative analysis of molecular targeted radiosensitizers in 2D and 3D cancer cell line models

**DOI:** 10.2340/1651-226X.2025.43916

**Published:** 2025-12-07

**Authors:** Michael Ramirez Parra, Antje Dietrich, Manuel Pfeifer, Henning Willers, Mechthild Krause, Nathalie Borgeaud

**Affiliations:** a OncoRay – National Center for Radiation Research in Oncology, Faculty of Medicine and University Hospital Carl Gustav Carus, TUD Dresden University of Technology, Helmholtz-Zentrum Dresden-Rossendorf, Dresden, Germany; b German Cancer Consortium (DKTK), Partner Site Dresden, and German Cancer Research Center (DKFZ), Heidelberg, Germany; c Institute of Legal Medicine, Faculty of Medicine, Technische Universität Dresden, Dresden, Germany; d Department of Radiation Oncology, Massachusetts General Hospital, Harvard Medical School, Boston, MA, USA; e National Center for Tumor Diseases (NCT), NCT/UCC Dresden, A Partnership Between German Cancer Research Center (DKFZ), Faculty of Medicine and University Hospital Carl Gustav Carus, TUD Dresden University of Technology, and Helmholtz-Zentrum Dresden-Rossendorf (HZDR), Germany; f Department of Radiotherapy and Radiation Oncology, Faculty of Medicine and University Hospital Carl Gustav Carus, TUD Dresden University of Technology, Dresden, Germany; g Helmholtz-Zentrum Dresden-Rossendorf, Dresden, Germany

**Keywords:** 3D cultures, non-small cell lung cancer, DNA-PK, ATR, PARP, IAP

## Abstract

**Background and purpose:**

Despite being a critical treatment modality, radiotherapy effectiveness is often limited by tumor resistance. Therefore, there exists a need to identify molecular targeted drugs that enhance the therapeutic response to radiation. We hypothesize that a systematic comparison of targeted radiosensitizers across two-dimensional (2D) and three-dimensional (3D) spheroid cultures will reveal context-specific differences in radiosensitivity to guide preclinical prioritization of candidate radiosensitizers.

**Material and methods:**

Radiosensitizing effects of DNA-PKcs (M3814), ATR (M6620), PARP (Olaparib), and IAP (Birinapant) inhibitors using a panel of lung cancer cell lines were studied. A 3D extracellular matrix (ECM) colony formation assay for single doses of 0–6 Gy, coupled with automated colony counting, was implemented alongside standard 2D colony formation assays. Dose Enhancement Factor (DEF_0.1SF_) was used to compare radiosensitizing effects, and drug–radiation interactions were assessed using the Synergyfinder tool.

**Results:**

DNA-PKcs and ATR inhibitors induced moderate to strong dose-dependent radiosensitization (DEF_0.1SF_ > 1.4 for at least one drug concentration) in most cell lines under both conditions (15/16 drug/cell line combinations). PARP inhibition showed similar effects in 3D and 2D (2/3 vs 3/5 combinations). Birinapant showed no relevant effect. The strongest synergy was at 2 Gy, particularly with the DNA-PK inhibitor in both culture models.

**Interpretation:**

Integrating multiple culture models enhances the detection of cell line – and drug-specific radiosensitization. Although 2D and 3D cultures produced largely similar results, and 2D assays provide a practical alternative when 3D methods are not feasible, the 3D cultures reveal additional ECM-dependent responses. These results emphasize the utility of physiologically relevant platforms for robust screening and prioritization of candidate radiosensitizers.

## Introduction

Precision medicine strategies, using molecular targeted agents to sensitize tumor cells to radiation, offer a promising approach to improve clinical outcomes of radiotherapy [1, 2]. However, the translation of targeted radiosensitizers into clinical practice remains challenging due to limitations of traditional preclinical models, including problems with assay reproducibility and the use of culture platforms that do not accurately capture clinically relevant tumor heterogeneity and microenvironmental influences [3]. Therefore, employing physiologically relevant platforms, such as three-dimensional (3D) cultures, that better recapitulate tumor architecture, nutrient gradients, and extracellular matrix (ECM) interactions, known to regulate radioresistance [4–6], may refine the process of identifying and prioritizing radiosensitizers with clinical potential.

We hypothesize that specific targeted inhibitors enhance radiation response in molecularly distinct subsets of lung cancer cells, and that a systematic comparison across two-dimensional (2D) and 3D spheroid cultures will reveal context-dependent differences in radiosensitivity which may guide prioritization of candidate radiosensitizers for preclinical development.

In this study, by exploring the radiosensitizing effects of four molecular therapies targeting DNA repair (M3814, M6620, Olaparib) and apoptotic (Birinapant) pathways in 2D and 3D cultures, we aim to reveal culture related differences that can guide the prioritization of radiosensitizers in preclinical assays. We selected genomically diverse non-small cell lung cancer (NSCLC) cell lines as models given the need to develop novel combination therapies in this disease [2]. Our approach adhered to established recommendations for ensuring reproducibility in preclinical experiments involving drug–radiation combinations [3]. Our data demonstrate potentially important similarities and differences of targeted radiosensitizer effects in 2D versus 3D cultures, and we identify specific doses of targeted drugs that exhibit strong synergy with radiotherapy, particularly at clinical standard doses of 2 Gy.

## Material and methods

### Cell culture and treatments

A panel of genomically diverse cell lines of NSCLC representing the different histological subtypes defined for this disease: Adenocarcinoma (NCI-H23, NCI-H441, Calu-6, and SW1573), Squamous Cell Carcinoma (NCI-H1703), and Large Cell Carcinoma (NCI-H460) are from the American Type Culture Collection. Cell line authentication using Short Tandem Repeat (STR) profiling was performed with the PowerPlex ESX17 Fast System® (Promega) and capillary electrophoresis (Genetic Analyzer 3500) according to the manufacturer’s instructions.

NCI-H460, NCI-H441, Calu-6, and NCI-H1703 cells were maintained in RPMI-1640 (Sigma (Merck), R2405), SW1573 cells in Advanced Dubelcco’s Modified Eagles Medium (DMEM)/F12 (Gibco, 12634-01), and NCI-H23 cells in DMEM (Sigma, D0819) supplemented with 10% Fetal Calf serum (Sigma, F7524) and 1% Penicillin Streptomycin (Sigma, P0781) at 37°C with 5% CO_2_ and humidified atmosphere.

The four inhibitors targeting DNA-repair (DNA-PKi M3814, ATRi M6620, PARPi Olaparib) and apoptotic pathways (IAPi Birinapant) were among the most active agents in a previously performed screen of 3D NSCLC cultures [2]. Details for the inhibitors are given in Table 1. 2D viability assays were conducted to determine inhibitor concentrations for radiosensitization experiments (Supplementary Figure 1). Two to three concentrations were selected for the colony formation assays (Table 1), which reduced cell viability by no more than 60%, ensuring moderate single-agent cellular toxicity.

**Table 1:** List of inhibitors and concentrations used in colony formation assays.

Drug	Company	Name/Lot etc.	Concentrations
**DNA-PKi**	Biomol	M3814, Nedisertib, Peposertib Lot: LKT-M003814.5	18–200 (nM)
**ATRi**	APExBIO Technology	M6620, VE-822, Berzosertib Lot: B1383	18–200 (nM)
**PARPi**	Cayman chemical	Olaparib/, Lot:10621	0.9–10 (μM)
**IAPi**	Selleckchem	Birinapant, Lot:TL3 2711	30–1000 (nM)

* All drugs were dissolved in DMSO (dimethyl sulfoxide) (Sigma, 472301-1L).

Irradiation (0–8 Gy for intrinsic radiosensitivity; 0–6 Gy for radiosensitization assays) was delivered as single doses of 200 kV X-ray, 20 mA filtered with 0.5 mm Cu at a dose-rate of ~1.3 Gy/min (Yxlon Y.TU 320). Daily dosimetry was performed for quality assurance.

### Colony formation assays

For 2D experiments, single cells were seeded in 12-well plates. After 24 hours, cells were treated with inhibitors (Table 1) and irradiated 3 hours later. After another 24 hours incubation, drugs were washed off, and fresh medium was added. Cells were cultured for 7–18 days depending on cell line doubling time; then colonies were fixed in 70% ethanol, stained (Coomassie blue), and manually counted (Zeiss stemi 508 stereomicroscope). Cell clusters of over 50 cells were considered surviving colonies.

The 3D clonogenic assay was performed as described [5]. Single cells were mixed with medium containing 0.5 mg/ml Matrigel and seeded into 96-well plates coated with 1% agarose. After 24 hours, a medium with inhibitors (Table 1) was added and incubated for another 24 hours. Cells were irradiated and incubated for 8–15 days. Colonies were fixed and stained with 2% paraformaldehyde in PBS containing Hoechst (1:1000) overnight at 4°C. Wells were imaged using the Cytation 5 reader with 10 Z stacks at 90 µm intervals using a 4× objective and DAPI (4’,6-diamidino-2-phenylindole) filter. Images were processed with focus stacking and auto-stitching (Gen5 software, BioTek). Colony segmentation was done using the Stardist plugin [7] and counted with a semi-automated workflow in Fiji software. The size distribution was obtained using the ‘analyze particles’ function. The minimum colony size corresponding to more than 50 cells was determined visually, with 3D colonies above the cut-off diameter (NCI-H460 90 µm, NCI-H441, and Calu-6 120 µm) considered as surviving colonies.

Surviving fractions were normalized with the plating efficiency of drug-only controls as published [25]. Curves were fitted using the linear-quadratic (LQ) model [26] and the dose corresponding to 10% survival (SF0.1) was derived from these LQ fits. Dose enhancement factors at 10% surviving fraction (DEF_SF0.1_) were calculated using the following formula:


$$\text{DEF SF0.1 = }\frac{\text{Dose at SF0.1 (radiation + inhibitor)}}{\text{Dose at SF0.1 radiation only}}$$

Fittings and calculations were done with Graphpad Prism 8.0 software (Graphpad Software, San Diego, CA, USA). According to the DEF_SF0.1_ values, we arbitrarily defined the degree of radiosensitization as weak (1.0–1.1), mild (1.2–1.3), moderate (1.4–1.8), and strong (> 1.8).

### Synergy analysis

Surviving fractions were normalized to untreated controls and used to create dose–response matrices for each combination of inhibitor and radiation dose. The Zero Interaction Potency (ZIP) model synergy scores were calculated using the SynergyFinder framework [10]. Briefly, this tool is based on the dose–effect approach and compares changes in drug potency of the dose–response curves between single and combined drugs. ZIP scores indicate the excess response expected from radiation–drug interactions. For example, a ZIP score of 20 indicates a 20% greater effect in the combination treatment [11] Combinations with ZIP scores > 10 (i.e. 10%) were considered synergistic, and those between –10 and 10 were additive.

### Statistical analysis

Differences between radiation dose–response curves of treated and control (0 nM) cells were analyzed using an LQ model including treatment–dose interaction terms. The significance of differences in α and β parameters between groups was assessed by comparing full and reduced models using an analysis of variance (*F*-test) with the statistical package for the social sciences (SPSS) v23 [12]. *P* values ≤ 0.05 were considered statistically significant. Data presented correspond to the mean with standard deviation of at least three independent experiments in triplicate.

## Results

### Characterization and response of NSCLC cell lines to radiation and targeted inhibitors in 2D and 3D models

**Figure 1:**
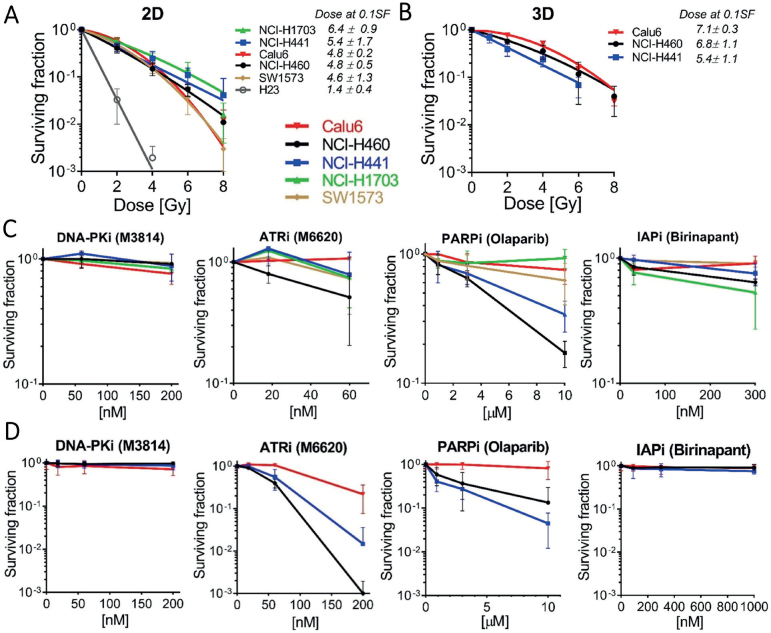
Response of non-small cell lung cancer (NSCLC) cell lines to single effects of radiation and targeted inhibitors in two-dimensional (2D) and three-dimensional (3D) models. Cell lines were irradiated with 0–8 Gy and colony formation was assessed under (A) 2D and (B) 3D culture conditions. Doses at 0.1SF ± SD are indicated. Survival curves (normalized to untreated DMSO controls) of cell lines treated with increasing doses of DNA-PKi (M3814), ATRi (M6620), PARPi (Olaparib), and IAPi (Birinapant) in (C) 2D or (D) 3D cultures are shown. Graphs represent the mean surviving fraction ± SD of three independent experiments in triplicate. SF: surviving fraction.

We evaluated the intrinsic radiosensitivity of NSCLC-derived cell lines in both 2D and 3D setups. Clonogenic assays were performed on the entire panel in 2D conditions, as shown in Figure 1A. For the 3D NSCLC panel, only three cell lines were included due to intense aggregation and poor colony formation observed for NCI-H23, NCI-H1703, and SW1573 cells (Figure 1B). In the 2D condition, we found the NCI-H23 model to be the most radiosensitive of the panel, with only 3.3% survival at 2 Gy. Therefore, it was excluded from further experiments. Radiation doses at SF_0.1_ (D_0.1_) were calculated from the LQ regression. We found SW1573, NCI-H460, and Calu-6 to be moderately radiosensitive (4.6 ± 1.3, 4.8 ± 0.5, and 4.8 ± 0.2 Gy, respectively) and NCI-H441 (5.4 ± 1.7 Gy) and NCI-H1703 (6.4 ± 0.9 Gy) to be the most radioresistant lines (Figure 1A). In the 3D condition, NCI-H460 and Calu-6 exhibited similar radiosensitivity when comparing radiation dose at SF_0.1_ (6.8 ± 1.1 Gy and 7.1 ± 0.3 Gy respectively) (Figure 1B). Interestingly, we observed a shift towards radioresistance in the 3D conditions for NCI-H460 and Calu-6 when compared to their respective counterparts in the 2D assays (Fold change of D_0.1_ = 1.4 and 1.5 respectively) in contrast to NCI-H441 (Fold change D_0.1_ = 1.0).

We next performed clonogenic assays to determine the effects of the inhibitors as single agents. Under 2D conditions, the surviving fractions were above 60% with all inhibitors except for IAPi in NCI-H1703, ATRi in NCI-H460, and PARPi in NCI-H441 and NCI-H460 reducing the surviving fraction by about 50% or more at the highest doses, respectively (Figure 1C). Interestingly, Calu-6 showed resistance to single drug effects with all inhibitors in 2D. In 3D conditions, the DNA-PKi and IAPi did not significantly reduce the surviving fractions in any of the cell lines. However, we observed a dose-dependent reduction in the surviving fractions of NCI-H460 and NCI-H441 when treated with PARPi and ATRi, respectively (Figure 1D).

### Radiosensitizing potential of targeted inhibitors in 2D NSCLC models

**Figure 2:**
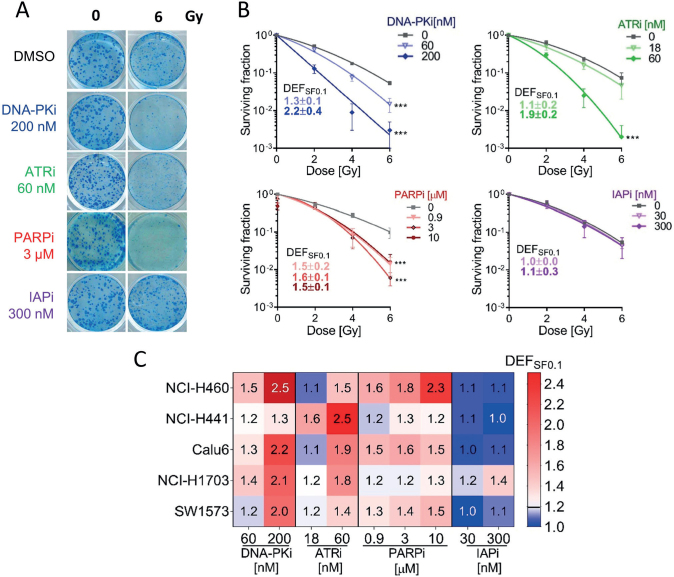
Sensitivity of non-small cell lung cancer (NSCLC) cell lines to targeted inhibitors in combination with radiation in two-dimensional (2D) cultures. (A) Representative images of Calu-6 colonies after treatment with targeted inhibitors combined with 6 Gy of radiation. (B) Surviving fraction plots of Calu-6 cells treated with increasing doses of targeted inhibitors combined with 0–6 Gy doses of radiation. Data were fitted with the linear-quadratic (LQ) model and Dose enhancement factors at 0.1 surviving fractions (DEF_0.1SF_) were calculated. Data points correspond to mean ± SD of three independent experiments in triplicate. (C) Heatmap representing the DEF_0.1SF_ calculated from surviving plots from the full set of NSCLC cell lines evaluated. Statistical significance was evaluated on LQ regressions using an *F*-test. *p < 0.05, **p < 0.01, ***p < 0.001.

To evaluate the radiosensitizing effects of targeted inhibitors across the NSCLC panel, we conducted colony formation assays using cells treated with increasing drug concentrations, with or without radiation. DEF_SF0.1_ for each inhibitor dose is shown in Supplementary Figure 2. Representative images of colonies after treatments in the Calu-6 model are shown in Figure 2A. In this cell line, the DNA-PKi and ATRi were considered strong radiosensitizers, respectively, at the highest doses (DEF_SF0.1_ 2.2 ± 0.4 and 1.9 ± 0.2). In addition, PARPi was considered a moderate radiosensitizer (DEF_SF0.1_ 1.6 ± 0.1 at 3 μM) and IAPi did not have any effect (Figure 2B). Across the entire panel, we found that the effects of the inhibitors varied among cell lines. The DNA-PKi produced moderate to strong radiosensitization with a dose-dependent increase in DEF_SF0.1_ in all cell lines except for NCI-H441 (DEF_SF0.1_ = 1.3 ± 0.4 at 200 nM) (Figure 2C and Supplementary Figure 2). The PARPi showed a dose-dependent radiosensitization, with the strongest effects at 10 µM in NCI-H460 (DEF_SF0.1_ = 2.3 ± 0.4 at 10 µM) and moderate sensitization in SW1573 (DEF_SF0.1_ = 1.5 ± 0.2). The ATRi demonstrated global radiosensitization effects with the strongest effect in NCI-H441 (DEF_SF0.1_ = 2.5 ± 0.6 at 60 nM) and moderate radiosensitization in the other models (Figure 2C). In summary, DNA-PKi, and ATRi showed robust radiosensitizing effects across the entire panel in 2D conditions, while the PARPi moderate effects were cell-dependent, and IAPi demonstrated limited efficacy.

### Evaluation of radiosensitization in 3D NSCLC models

**Figure 3:**
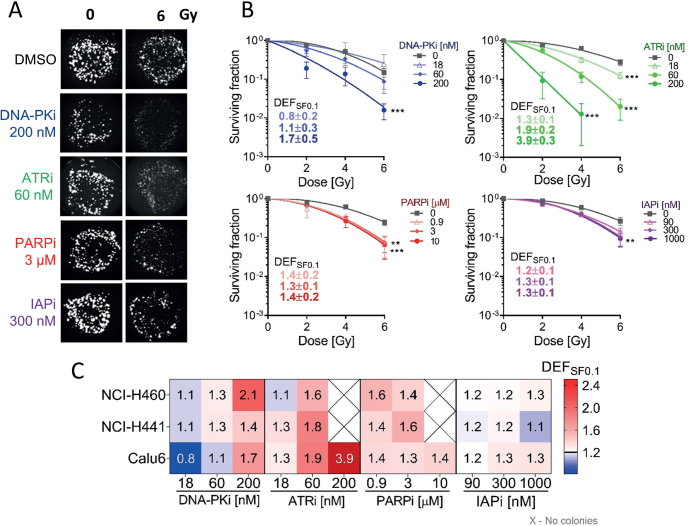
Radiosensitization profile of targeted inhibitors in combination with radiation in extracellular matrix-based three-dimensional (3D) cultures of non-small cell lung cancer (NSCLC) cell lines. (A) Representative images of Calu-6 3D colonies after treatment with targeted inhibitors combined with 6 Gy of radiation. (B) Surviving fraction plots of Calu-6 3D model treated with increasing doses of four targeted inhibitors combined with 0–6 Gy doses of radiation. Data were fitted with the linear-quadratic (LQ) model and dose enhancement factors at 0.1 surviving fractions (DEF_0.1SF_) were calculated. Data points correspond to mean ± SD of three independent experiments in triplicate. (C) Heat map representing the DEF_0.1SF_ calculated from surviving plots from the full set of NSCLC cell lines evaluated. Statistical significance was evaluated on LQ regression lines using an *F*-test. *p < 0.05, **p < 0.01, ***p < 0.001.

Representative images of 3D colonies and surviving fraction plots after treatments are exemplified for Calu-6 (Figure 3A). The results for all cell lines, shown in Supplementary Figure 3, revealed that DNA-PKi and ATRi induced moderate to strong, dose-dependent radiosensitization (DEF_SF0.1_ 1.4–2.0 at the highest drug concentration) across all models. Notably, the strongest radiosensitization was observed in Calu-6 cells with the ATRi (DEF_SF0.1_ = 3.9 ± 0.3 at 200 nM) (Figure 3B). We did not observe any radiosensitizing effect of the IAPi across the entire panel, except for a significant mild effect in 3D-Calu-6 (Figure 3B). Generally, the radiosensitization profiles of the inhibitors in 3D conditions showed similar trends to those observed in 2D cultures. Interestingly, in the NCI-H441 cells, the PARPi demonstrated a shift from weak/mild radiosensitization in 2D to moderate radiosensitization in 3D conditions at 0.9 and 3 μM (Figures 2C and 3C).

### Synergistic and additive effects of inhibitors with radiation in 2D and 3D NSCLC models

**Figure 4:**
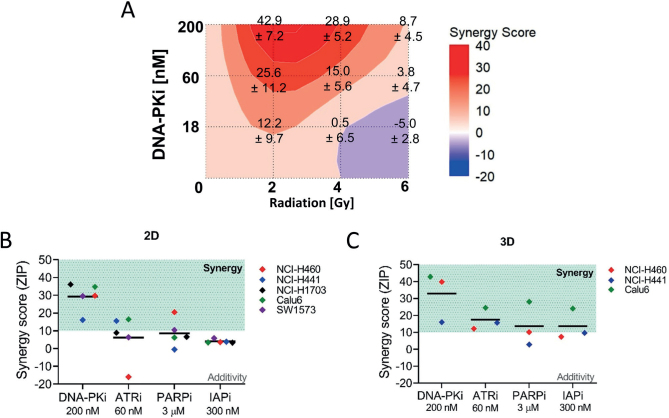
Synergistic interactions of targeted inhibitors with clinical doses of radiation in two-dimensional (2D) and three-dimensional (3D) models of non-small cell lung cancer (NSCLC) cell lines. (A) Zero Interaction Potency (ZIP) score matrix obtained as output from the Synergyfinder3.0 online tool. Representative image from 3D-Calu-6 model treated with DNA-PK inhibitor. Numbers indicate the mean ZIP score ± SD for each drug dose/radiation combination. (B) 2D and (C) 3D synergy score plots when combining the indicated doses of targeted inhibitors with 2 Gy radiation. Combinations with ZIP scores > 10 are considered synergistic and those between –10 and 10 correspond to additive effects.

To further characterize the interaction between radiation and targeted inhibitors, we assessed whether the observed sensitization was due to synergistic or additive effects as exemplified for Calu-6 (2D) in Figure 4A. The complete set of results for 2D and 3D models is shown in Supplementary Figures 4 and 5, respectively. Our analysis revealed that the greatest synergistic effects were observed when inhibitors were combined with 2 Gy radiation and corresponding ZIP scores are summarized in Figure 4B and C for 2D and 3D conditions, respectively. Notably, the DNA-PKi 200 nM demonstrated the most synergistic increase in radiation efficacy when combined with 2 Gy in 2D (ZIP scores: 16.1 ± 6.6 in NCI-H441 to 36.1 ± 4.4 in NCI-H1703, Figure 4B) and 3D lines (ZIP scores: 16.1 ± 5.9 to 42.9 ± 7.2, Figure 4C), confirming its strong potential as a radiosensitizer in NSCLC. Interestingly, Calu-6 showed marked differences between 2D and 3D cultures (Figure 4B–C green dots). In 2D culture, we observed additive effects with PARPi and IAPi (ZIP scores: 6.2 ± 7.2 and 3.5 ± 13.7, respectively). However, in 3D conditions there was a switch to synergistic effects (ZIP scores: 28.1 ± 15.5 and 24.1 ± 15.7). These results correlated with the global radiosensitization observed in the Calu-6 model, which ranged from mild to strong in all inhibitors (Figure 3C).

## Discussion and conclusion

To prioritize promising drug candidates for clinical trials, methodologies that accurately assess the radiosensitizing potential of targeted agents are required. In the present study, we investigated the radiosensitizing ability of multiple doses of a panel of targeted drugs using the colony formation assay in both 2D and 3D cultures derived from NSCLC cell lines. According to guidelines for improving the data quality of preclinical assays in radiobiology, statistically significant Dose Modifying Factors values higher than 1.2 are considered good predictors for clinical success [3]. Based on this parameter we identified DNA-PK and ATR inhibitors as the strongest radiosensitizers. Both drugs showed mild to moderate enhancement seen across all NSCLC cell lines with no significant differences in radiosensitization for 2D versus 3D models. Validation in multiple cell lines is important to represent the histological subtypes and diverse mutational landscape of the disease, aiming at the identification of potential markers of response in early phases of preclinical research [1]. Our results suggest that both DNA-PK and ATR inhibitors can be considered global radiosensitizers and do not require a specific biomarker to be effective in lung cancer models.

Olaparib, a PARP1/2i, has demonstrated radiosensitization in various tumor types including brain, colorectal, pancreatic, head and neck, and lung cancer in preclinical models. In line with our findings, enhancement ratios between 1.1 and 2.9 in the micromolar range have been reported for this drug in vitro [13]. For the concentration range of Olaparib selected in our study, we only observed strong radiosensitization in 2D for the NCI-H460 model, while the rest of the models showed mild effects on our scoring scale. Interestingly, the PARPi demonstrated improved radiosensitization only in NCI-H441 in 3D conditions, highlighting the value of both 2D and 3D models in drug efficacy assessment

The SMAC mimetic drug Birinapant has been shown to induce apoptosis through the inhibition of IAP proteins and has demonstrated a sensitizing capacity in combination with radiation or chemotherapy in head and neck cancer [14, 15], and glioblastoma in vitro cancer models [8]. In this study, radiosensitizing effects of Birinapant were either absent or low in both 2D and 3D cultures. One possible explanation is an inactivation of NF-κB–dependent signalling, since prior studies showed that Birinapant was more effective in models with inducible TNFα/NF-κB responses after irradiation [8, 15]. However, since activation of canonical NF-κB signalling has been previously reported in NSCLC cells included in our panel [9], other factors are likely to contribute to the modest effects observed. Our data are consistent with a report on NSCLC showing that even at 10 µM concentration, Birinapant did not exhibit a relevant sensitization in NCI-H460 (DEF = 1.16 at 4 Gy) [16]. Sustained drug presence in the medium may be required to achieve effective and lasting inhibition of cIAP proteins. Therefore, removing the drug after 24 hours in 2D cultures may have compromised its radiosensitizing effects, whereas continuous exposure in 3D conditions revealed its activity as observed for Calu-6. Further investigation with this drug is required to determine whether the order of addition of treatments or the degree of target dependency might improve the radiosensitizing potential of IAP inhibition in NSCLC.

In recent years, there has been a shift towards the use of 3D cultures over traditional 2D cultures in preclinical assays. Physiological conditions of tumors in vivo, such as morphology, development of hypoxia and nutrient gradients, interactions with ECM, and intratumoral heterogeneity, are better recapitulated in 3D models [4, 6]. Moreover, it has also been reported that 3D cultures exhibit enhanced radioresistance mediated by integrin–ECM interactions compared to 2D cultures [17]. Consistent with this, we found that lung cancer cell lines were more radioresistant under 3D conditions compared to 2D conditions. We do not exclude the possibility that changes at the transcriptomic level could be involved in the differential response to drugs and radiation between the models, as it was recently described in a colon and bladder cancer model [18]. However, we noted a trend towards higher enhancement factors in 2D conditions despite the shorter incubation times with drugs. We hypothesize that due to the additional interactions present in the 3D environment, extended treatment durations and higher drug concentrations are required to fully capture the sensitizing properties of targeted therapies in this preclinical model.

Studies have demonstrated that Matrigel concentration critically affects NSCLC cell behaviour. Specifically, lower Matrigel concentrations (2 mg/ml) have been shown to promote migration, whereas higher concentrations (4 mg/ml) reduce migration by increasing cell attachment and matrix stiffness [19, 20]. Furthermore, it has recently been shown that ECM stiffness in NSCLC cells influences not only gene expression but also drug sensitivity, with softer matrices promoting proliferation and sensitivity to DNA-damaging agents, whereas stiffer conditions can favor resistance [21]. These findings suggest that variations in ECM stiffness could introduce bias in 3D radiosensitivity assays, highlighting the need for future studies integrating the interplay between ECM stiffness and response to targeted radiosensitizers.

We further characterized the interactions of the panel of targeted inhibitors with radiation, evaluating the degree of synergy or additivity using the ZIP model. This model demonstrates a better performance in identifying experimentally confirmed drug synergy, maintaining a low false positive rate in large-scale data sets [11]. Notably, the highest synergy was observed when inhibitors were combined with 2 Gy radiation across the panel of NSCLC cell lines in both 2D and 3D models. This suggests that these inhibitors may be particularly effective when used in conjunction with standard fractionated radiotherapy regimens. Hence, the synergy analysis in combination with radiosensitization results highlights the DNA-PKi as the most promising sensitizer for investigation in further preclinical trials. Since the ZIP model requires a sufficiently wide range of concentrations to capture maximum, minimum, and EC50 effects [11], the limited number of drug concentrations in this study represents a limitation for accurately estimating synergy. However, we emphasize that both measures provide complementary information: the DEF quantifies the magnitude of radiosensitization if an effect is present, whereas the ZIP score characterizes the nature of the interaction (synergistic or additive), considering the influence of single-agent effects. The observed additivity with ATR and PARP inhibitors is of interest as well. Independent actions of drugs that enhance the efficacy of radiation are desirable, as either a synergistic or an additive effect can improve clinical outcomes. This, in turn, contributes to the translation of new drugs into clinical practice [22]. Together, both metrics may guide optimal radiation–drug dose selection to improve antitumor efficacy and potentially reduce treatment related toxicity.

Several limitations of the study should be considered. Many cell lines exhibit poor colony formation under 3D conditions. This limitation can restrict the data available for proper comparison between the models and also limit the value of those assays, especially if a broad variety of lines should be used. For example, the squamous cell carcinoma subtype was not represented among the cell lines successfully established in 3D culture. Additionally, mechanistic studies and mutational analyses of the cell lines used, particularly under 3D-ECM conditions, are necessary to identify biomarkers of radiosensitization. Such biomarkers could better explain the differential responses observed among the cell lines and inhibitors. The impact of the tumor microenvironment and vasculature on radiosensitization should also be considered, as in vitro cell culture assays alone cannot assess the efficacy of agents influenced by these factors. Future studies should extend these findings to patient-derived 3D models, which resemble clinical behavior more closely. Patient-derived organoids from surgically resected lung tumors retain the histological and molecular features of the original tissue, enabling accurate drug response assessment [23]. Additionally, NSCLC tumoroids have recently been shown to predict individual responses to radiotherapy and chemotherapy, highlighting their translational potential [24].

Collectively, our findings highlight the complementary value of 2D and 3D culture systems for evaluating targeted radiosensitizers in NSCLC. While both models produced largely comparable effects, 3D cultures revealed additional ECM-dependent responses. These results emphasize the importance of refining physiologically relevant in vitro approaches for more robust prioritization of targeted radiosensitizers.

## Supplementary Material



## Data Availability

The data used to support the findings of this study are available from the corresponding author upon request.
