# Supplementary material for: Comparative analysis of molecular targeted radiosensitizers in 2D and 3D cancer cell line models
Source: Acta Oncol. 2025 Dec 7;64:43916. doi: 10.2340/1651-226X.2025.43916 (PMC12696963; doi:10.2340/1651-226X.2025.43916)
Supplement: Supplementary file 1 [file AO-64-43916-s1.pdf]

Supplementary material has been published as submitted. It has not been copyedited, or typeset by Acta Oncologica

Supplementary Figure 1. Selection of targeted inhibitor concentrations for combination experiments with radiation in NSCLC cell lines. 2D cultures of NSCLC cell lines were treated with increasing concentrations of targeted inhibitors for 24 hours. Cell viability was measured with Prestoblue assay (Thermofisher, A13261) and fluorescence was registered using a Cytation 5 multimode reader (Agilent, BioTek). Cell viability was calculated relative to DMSO controls. Data represents mean ± SD of two independent experiments in triplicate.

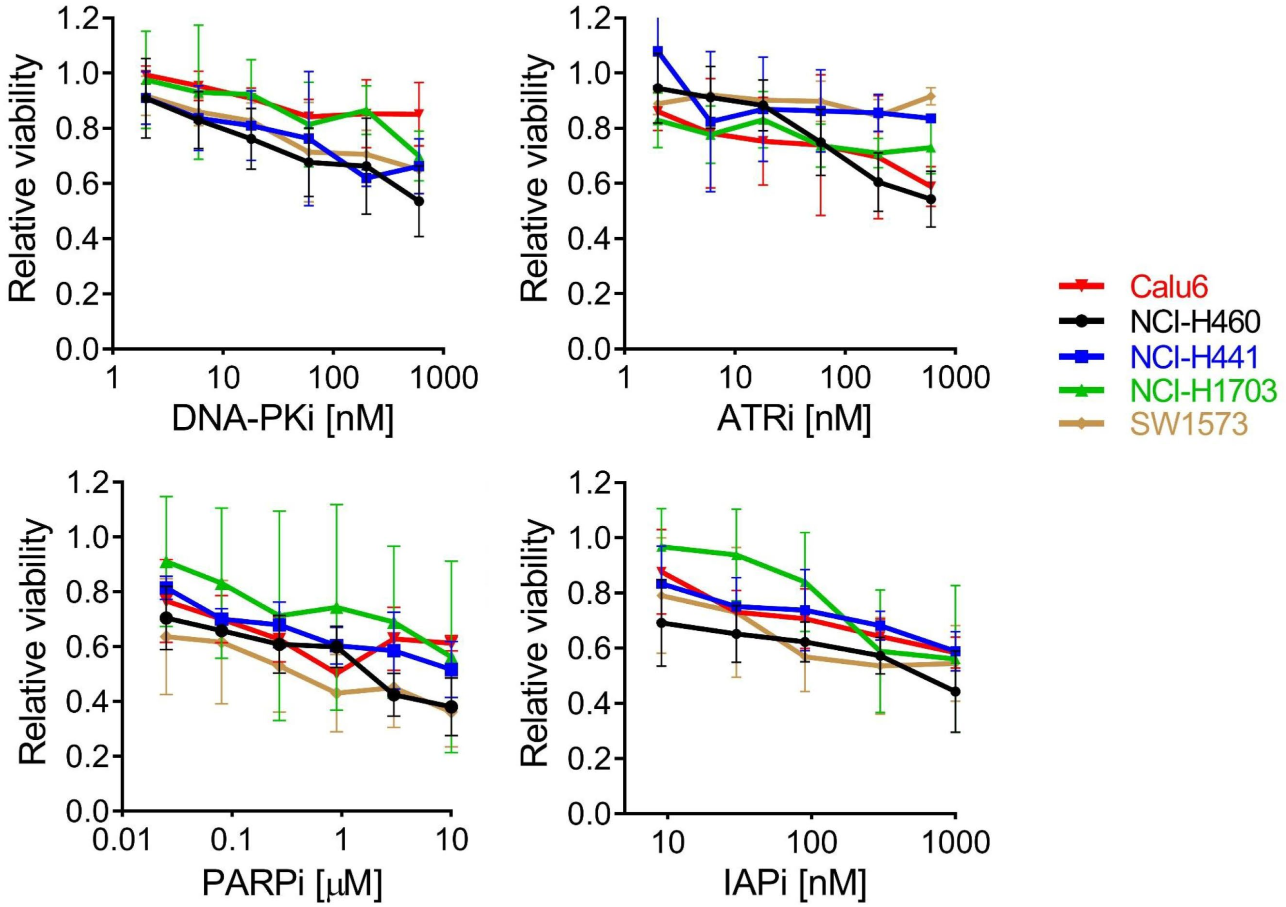

Supplementary Figure 2. Radiosensitization profile of a set of targeted drugs in the panel of NSCLC in 2D clonogenic assays. Cells were treated with increasing doses of targeted inhibitors for 24 hours and then irradiated with 0-6 Gy doses. Surviving fractions were fitted with the LQ regression and $DEF_{SF0.1}$ were calculated for each dose of inhibitor. Data points correspond to mean ± SD of three independent experiments in triplicate. Statistical significance was evaluated on LQ regression lines using an F-test. * $p < 0.05$, ** $p < 0.01$, *** $p < 0.001$

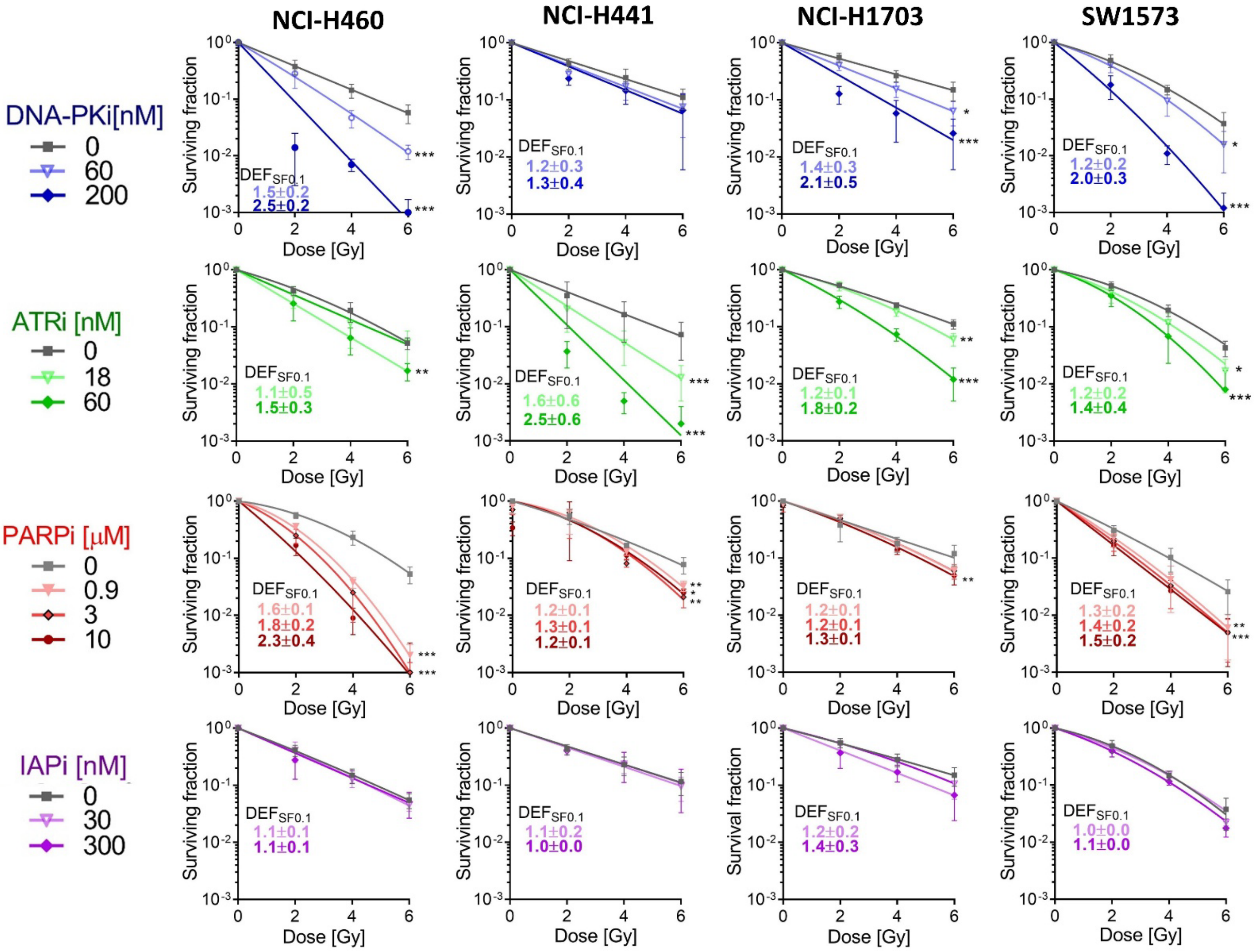

Supplementary Figure 3. Radiosensitization profile of a set of targeted drugs in the panel of NSCLC in 3D ECM-based clonogenic assays. Cells were treated with increasing doses of targeted inhibitors for 24 hours and then irradiated with 0-6 Gy doses. Surviving fractions were fitted with the LQ regression and $DEF_{SF0.1}$ were calculated for each dose of inhibitor. Data points correspond to mean ± SD of three independent experiments in triplicate. Missing lines represent conditions where 0 colonies were obtained at the end of the assay. Statistical significance was evaluated on LQ regression lines using an F-test. * $p < 0.05$, ** $p < 0.01$, *** $p < 0.001$.

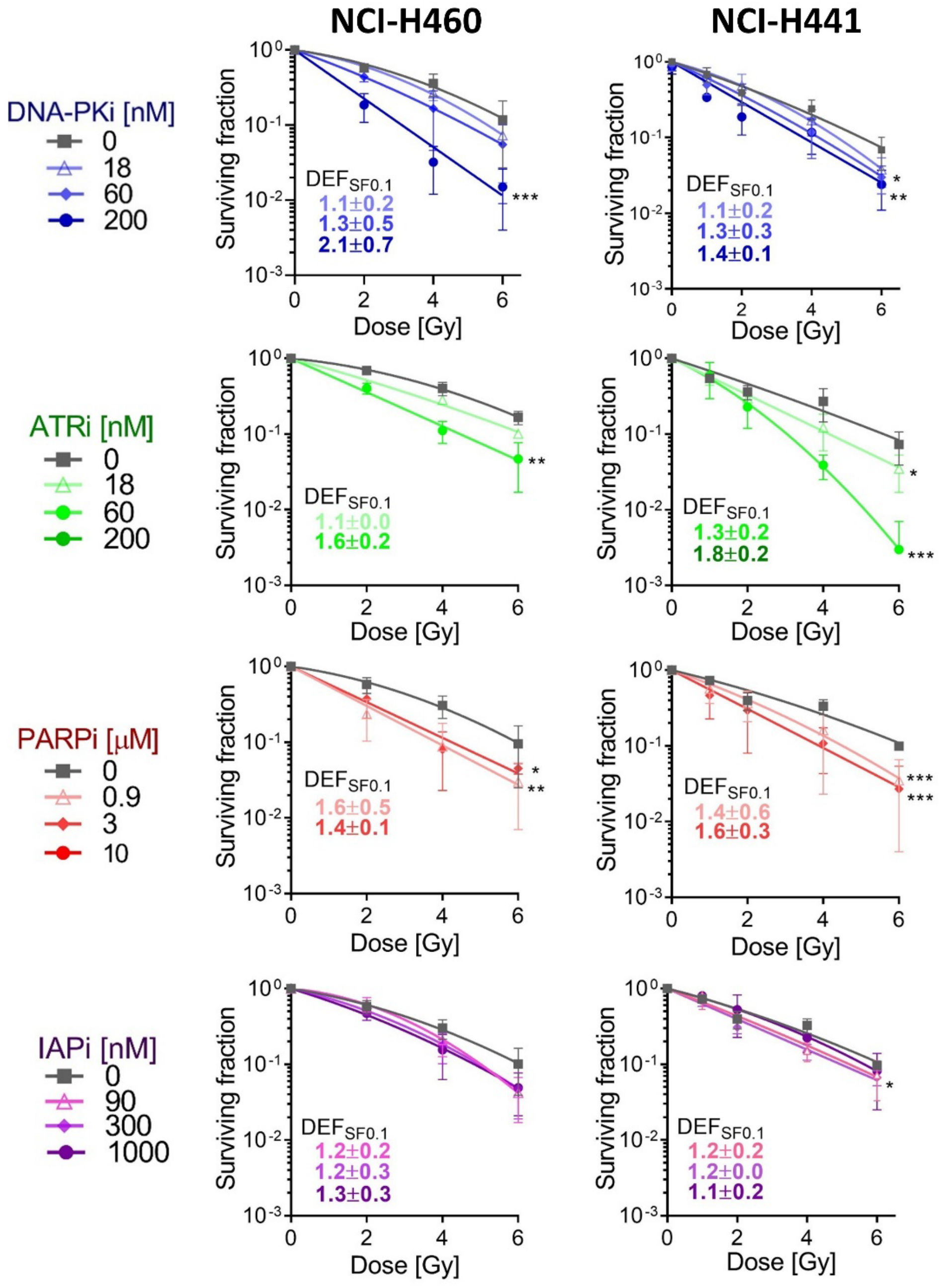

Supplementary Figure 4 Synergistic interactions between radiation and targeted inhibitors in 2D NSCLC cell lines. Surviving fractions (normalized to untreated controls) from 2D colony formation assays were reanalyzed with SynergyFinder 3.0 online tool using the ZIP synergy reference model. Bars represent mean ZIP score ± SD (n=3) for each drug dose/ radiation combination. Combinations with ZIP scores >10 were considered synergistic (Green background) and between -10 and to 10 were additive effects (White background).

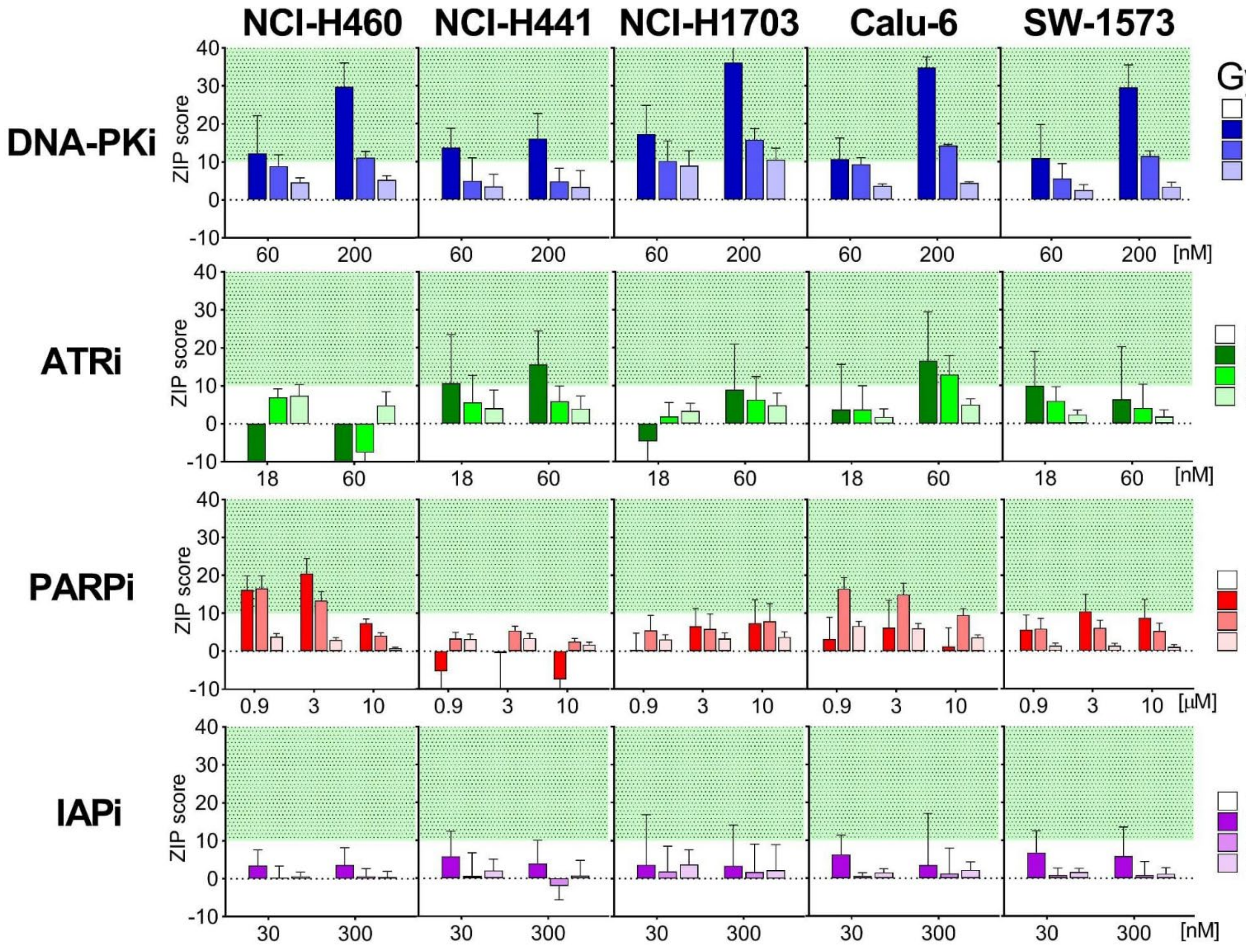

Supplementary Figure 5. Synergistic interactions between radiation and targeted inhibitors in 3D NSCLC cell lines. Absolute surviving fractions from 3D colony formation assays were reanalyzed with SynergyFinder 3.0 online tool using the ZIP synergy reference model. Bars represent mean ZIP score±SD (n=3) for each drug dose/ radiation combination. Combinations with ZIP scores >10 are considered as synergistic (Green background) and between -10 to 10 as additive effects (White background)

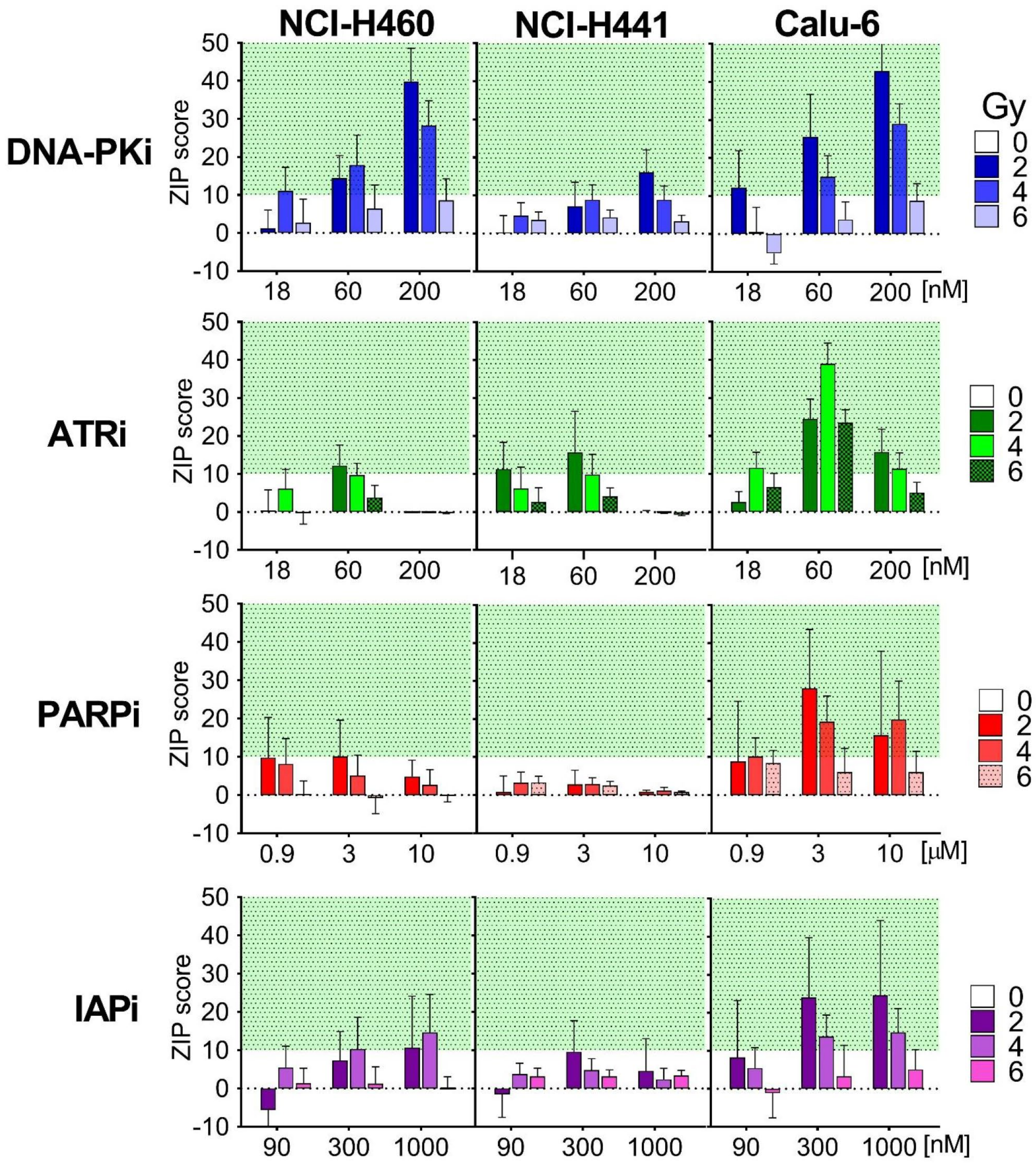

Supplementary Table 1. Summary of ZIP synergy scores (mean ± SD) of three independent experiments in 2D cultures following combined treatment with increasing inhibitor concentrations and 2 Gy irradiation. Data correspond to the numerical values of the plot shown in Figure 4B. Conc: drug concentration.

		Mean ZIP score ± SD				
**Inhibitor**	**Conc**	**NCI-H460**	**NCI-H441**	**NCI-H1703**	**Calu-6**	**SW1573**
**DNA-PKi [nM]**	60	12.3±9.9	13.7±5.2	17.3±7.6	10.7±5.6	11.0±8.8
	200	29.8±6.3	16.1±6.6	36.1±4.4	34.8±2.9	29.6±6.0
**ATRi [nM]**	18	-12.4±10.8	10.6±12.9	-4.6±7.7	3.7±11.9	10.0±9.1
	60	-16±4.6	15.6±8.8	8.9±12.0	16.5±12.9	6.4±13.8
**PARPi [µM]**	0.9	16.2±3.8	-5.3±7.7	0.3±4.4	3.2±5.7	5.6±3.9
	3	20.5±3.9	-0.5±10.7	6.6±4.7	6.2±7.2	10.5±4.5
	10	7.4±1.1	-7.5±10.6	7.4±6.2	1.2±4.9	8.7±4.8
**IAPi [nM]**	30	3.5±4.2	5.9±6.6	3.7±13.2	6.3±5.1	6.8±5.8
	300	3.7±4.6	3.9±6.2	3.3±10.8	3.5±13.7	5.9±7.7

Supplementary Table 2. Summary of ZIP synergy scores (mean ± SD) of three independent experiments in triplicates in 3D cultures following combined treatment with increasing inhibitor concentrations and 2 Gy irradiation. Data correspond to the numerical values of the plot shown in Figure 4C. Conc: drug concentration.

		Mean ZIP score ± SD		
**Inhibitor**	**Conc**	**NCI-H460**	**NCI-H441**	**Calu-6**
**DNA-PKi [nM]**	18	1.2±4.9	0.3±4.4	12.2±9.7
	60	14.4±6.0	7.2±6.3	25.6±11.2
	200	39.8±9.8	16.1±5.9	42.9±7.2
**ATRi [nM]**	18	0.4±5.4	11.3±7.1	2.7±2.7
	60	12.2±5.4	15.7±10.8	24.5±5.2
	200	0.0±0.1	0.0±0.4	15.8±6.0
**PARPi [µM]**	0.9	9.8±10.4	0.8±4.3	8.9±15.8
	3	10.1±9.4	2.8±3.8	28.1±15.5
	10	4.9±4.3	0.9±0.5	15.8±22.1
**IAPi [nM]**	90	-5.6±5.6	-1.5±6.0	8.3±15.0
	300	7.4±7.5	9.6±8.2	24.1±15.7
	1000	10.7±13.5	4.6±8.4	24.6±19.6
